# Inhibition of cancer-type amino acid transporter LAT1 suppresses B16-F10 melanoma metastasis in mouse models

**DOI:** 10.1038/s41598-023-41096-3

**Published:** 2023-08-25

**Authors:** Zitong Shi, Kazuko Kaneda-Nakashima, Ryuichi Ohgaki, Minhui Xu, Hiroki Okanishi, Hitoshi Endou, Shushi Nagamori, Yoshikatsu Kanai

**Affiliations:** 1 Department of Bio-System Pharmacology, Graduate School of Medicine, Osaka University, 2-2, Yamadaoka, Suita, Osaka 565-0871 Japan; 2 MS-CORE, FRC, Graduate School of Science, Osaka University, 1-1, Machikaneyama, Toyonaka, Osaka 560-0043 Japan; 3 Division of Science, Institute for Radiation Sciences, Osaka University, 2-4, Yamadaoka, Suita, Osaka 565-0871 Japan; 4 Integrated Frontier Research for Medical Science Division, Institute for Open and Transdisciplinary Research Initiatives, Osaka University, Suita, Osaka 565-0871 Japan; 5 J-Pharma Co., Ltd, Yokohama, Kanagawa 230-0046 Japan; 6 Center for SI Medical Research, The Jikei University School of Medicine, 3-25-8, Nishi-Shimbashi, Minato, Tokyo 105-8461 Japan; 7 Department of Laboratory Medicine, The Jikei University School of Medicine, 3-25-8, Nishi-Shimbashi, Minato, Tokyo 105-8461 Japan

**Keywords:** Cancer, Molecular biology, Oncology

## Abstract

Metastasis is the leading cause of mortality in cancer patients. L-type amino acid transporter 1 (LAT1, SLC7A5) is a Na^+^-independent neutral amino acid transporter highly expressed in various cancers to support their growth. Although high LAT1 expression is closely associated with cancer metastasis, its role in this process remains unclear. This study aimed to investigate the effect of LAT1 inhibition on cancer metastasis using B16-F10 melanoma mouse models. Our results demonstrated that nanvuranlat (JPH203), a high-affinity LAT1-selective inhibitor, suppressed B16-F10 cell proliferation, migration, and invasion. Similarly, LAT1 knockdown reduced cell proliferation, migration, and invasion. LAT1 inhibitors and LAT1 knockdown diminished B16-F10 lung metastasis in a lung metastasis model. Furthermore, nanvuranlat and LAT1 knockdown suppressed lung, spleen, and lymph node metastasis in an orthotopic metastasis model. We discovered that the LAT1 inhibitor reduced the cell surface expression of integrin αvβ3. Our findings revealed that the downregulation of the mTOR signaling pathway, induced by LAT1 inhibitors, decreased the expression of integrin αvβ3, contributing to the suppression of metastasis. These results highlight the critical role of LAT1 in cancer metastasis and suggest that LAT1 inhibition may serve as a potential target for anti-metastasis cancer therapy.

## Introduction

Cancer cells require high nutrients, including sugars and amino acids, to maintain their rapid proliferation. The cellular uptake of amino acids relies on amino acid transporters on the plasma membrane^1^. Cancer cells take up amino acids through amino acid transporters, including System L, an amino acid transport system that mediates the Na^+^-independent transport of large neutral amino acids^1^. System L is currently known to consist of four isoforms: LAT1 (SLC7A5), LAT2 (SLC7A8), LAT3 (SLC43A1), and LAT4 (SLC43A2)^2^. LAT1 and LAT2 each form a heterodimer with 4F2hc (SLC3A2) via disulfide bonds to be functional, while LAT3 and LAT4 function as monomers^2^.

Among the four System L isoforms, LAT1 is highly expressed in various cancer cells, including non-small cell lung cancer, liver cancer, triple-negative breast cancer, pancreatic ductal adenocarcinomas, biliary tract cancer, prostate cancer, and melanoma^2^. High LAT1 expression has been identified as a significant independent factor suggesting poor prognosis^3–8^. Due to its high expression in cancer cells, LAT1 is considered a potential target for cancer therapy^2, 9, 10^. Inhibition of LAT1 could suppress cancer cell growth by depriving cancer cells of essential amino acids^2^. The classic System L inhibitor, 2-aminobicyclo[2.2.1]heptane-2-carboxylic acid (BCH), despite its low affinity and limited selectivity for LAT1, has been shown to inhibit the growth of cancer cells^11, 12^. Nanvuranlat (JPH203) was developed as a high-affinity LAT1-selective inhibitor^2, 13^. Studies have demonstrated that nanvuranlat inhibits the growth of various cancer cell lines in vitro^13–15^ and suppresses the growth of xenograft tumors of cholangiocarcinoma^16^, pancreatic cancer^17^, and colorectal cancer^13^, suggesting that nanvuranlat could be a novel anti-cancer agent. The phase II clinical trial met the primary endpoint and demonstrated positive clinical efficacy in patients with biliary tract cancer^18^.

Metastasis is the leading cause of cancer-related deaths, accounting for over 90% of cancer-related fatalities^19^. Therefore, developing new therapeutic strategies for anti-metastasis treatment is urgently needed. Cancer metastasis involves the following processes: cancer cells first undergo an epithelial-mesenchymal transition (EMT) at the primary site, resulting in the loss of cell–cell adhesion and acquisition of migratory and invasive properties^20^. Then, cancer cells invade blood or lymphatic vessels, move into the circulatory systems, escape from blood and lymphatic vessels at a distant site, and colonize the metastatic site^20^. Common metastatic sites include lymph nodes, liver, lung, bone, and spleen^21^. High LAT1 expression has been correlated with lymphatic metastasis in biliary tract cancer^3^, pancreatic ductal adenocarcinoma^22^, and non-small cell lung cancer^7^, suggesting that LAT1 could be considered a potential target for anti-metastasis treatment. However, few studies have addressed the role of LAT1 in cancer metastasis.

Metastasis models using mouse B16-F10 melanoma cells are commonly applied in experimental research to examine the effect on cancer metastasis^23^. By injecting B16-F10 cells into the tail vein of C57BL/6 mice, tumor cells travel through the bloodstream to the lungs, forming metastatic nodules^23^. However, this tail vein injection-lung metastasis model does not include the process of cell migration and invasion at the primary site^24^. Furthermore, the number of injected cells in this model is greater than that of cancer cells typically present in circulation during natural metastasis, which constrains the model's capacity to represent the entire metastasis process accurately^24^. To address this limitation, we also employed an orthotopic metastasis model by injecting B16-F10 cells into the footpad of mice. In this model, sentinel lymph nodes are the first sites reached by metastatic cells, and metastasis in distant organs involves both hematogenous and lymphatic spread^25^.

In this study, we utilized the B16-F10 model to investigate the role of LAT1 in cancer metastasis. We initially discovered that LAT1 inhibitors and LAT1 knockdown reduced B16-F10 cell migration and invasion in vitro. In vivo experiments demonstrated that LAT1 inhibitors and LAT1 knockdown suppressed B16-F10 metastasis in the tail vein injection-lung metastasis model. Furthermore, B16-F10 metastasis in the lung, spleen, and lymph nodes was diminished by LAT1 inhibition in the orthotopic model. Flow cytometry analysis results suggested that decreased expression of integrin αvβ3, induced by LAT1 inhibition, might contribute to the suppression of cancer metastasis.

## Results

### Inhibition of LAT1 suppresses B16-F10 cell proliferation and tumor growth

**Figure 1:**
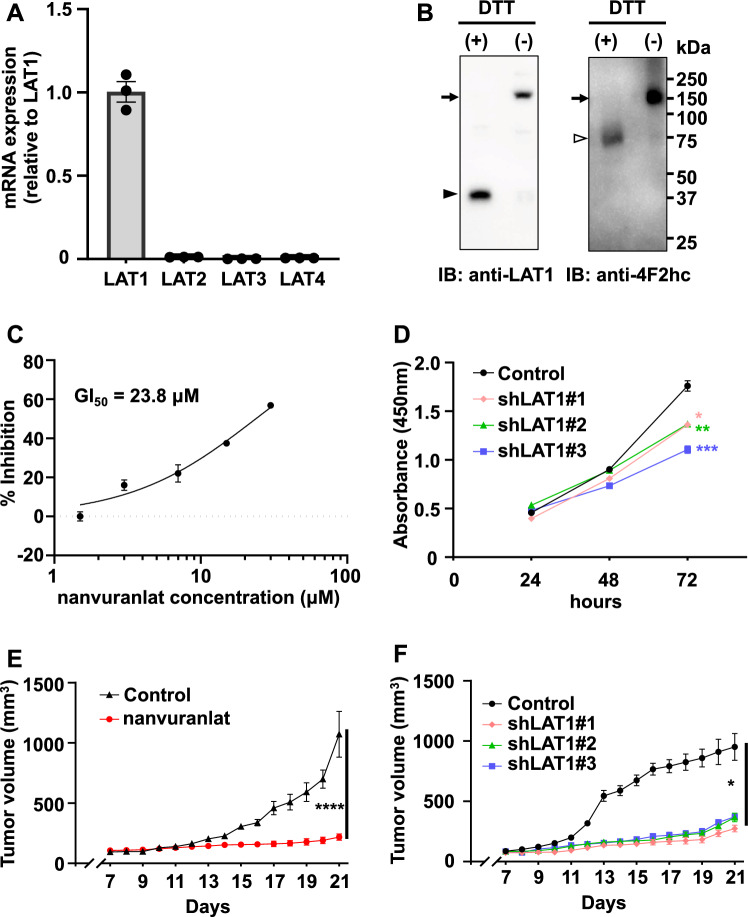
Expression of LAT1 in B16-F10 melanoma cells and the effect of LAT1 suppression on cell proliferation and tumor growth. (**A**) Quantification of LAT1, LAT2, LAT3, and LAT4 mRNA levels by RT-qPCR. Data were normalized to the expression of GAPDH mRNA and shown as relative expression levels compared to LAT1. PCR analyses were performed in triplicate (n = 3) and shown as mean ± SEM. (**B**) Protein expression of LAT1 and 4F2hc in B16-F10 cells. LAT1 and 4F2hc were detected by western blot using an anti-LAT1 antibody (left panel) and anti-4F2hc antibody (right panel), respectively, under reducing (DTT( +)) and non-reducing (DTT(-)) conditions. Arrows indicate the bands corresponding to LAT1-4F2hc heterodimer at 150 kDa. The bands of LAT1 (37 kDa) and 4F2hc (70 kDa) monomers are indicated by black and white arrowheads, respectively. Original, uncropped electrophoretic blots are presented in Supplementary Fig. S5. (**C**) Concentration-inhibition curve of nanvuranlat. B16-F10 cells were treated with nanvuranlat (1.5, 3, 7, 15, and 30 μM) for 48 h. The concentration-inhibition curve shows the percentage of inhibition of B16-F10 cell growth versus the concentration of nanvuranlat (n = 4, data are mean ± SEM). The GI_50_ value was calculated by nonlinear regression analysis: GI_50_ = 23.8 ± 3.6 μM. (**D**) Suppression of cell proliferation in B16-F10 LAT1-knockdown cells. Cell proliferation of B16-F10 LAT1-knockdown cells (shLAT1#1, #2, and #3) and control cells (B16-F10 cells transfected with control shRNA) was determined by the absorbance at 450 nm in the CCK-8 assay and shown at 24, 48, and 72 h after cell seeding. (**E**) Inhibitory effect of nanvuranlat on tumor growth compared with placebo control. On Day 0, B16-F10 cells were transplanted subcutaneously into the footpad of the hind leg of the mice. Nanvuranlat administration (i.v., 25 mg/kg, daily) started on Day 7 and finished on Day 20. Tumor size was measured from Day 7 to Day 21. (**F**) Suppression of B16-F10 tumor growth by LAT1 knockdown. Statistical significance was determined using two-way ANOVA with Sidak's post-test (n = 5, * *p* < 0.05, ** *p* < 0.01, *** *p* < 0.001, **** *p* < 0.0001). Data are mean ± SEM.

We first performed RT-qPCR to examine the expression levels of System L transporters in the B16-F10 melanoma cell line. Results showed that LAT1 was expressed in B16-F10 cells, while LAT2, LAT3, and LAT4 were not detected (Fig. 1A). We then investigated the expression of LAT1 protein by western blot analysis. The levels of LAT1 protein expression and 4F2hc in B16-F10 cells were examined under reducing (DTT(+)) and non-reducing (DTT(−)) conditions. In non-reducing conditions, a band corresponding to the LAT1-4F2hc heterodimer was observed at 150 kDa. In reducing conditions, bands of LAT1 and 4F2hc monomers were observed at 37 and 70 kDa, respectively (Fig. 1B). Next, we investigated the role of LAT1 in the proliferation of B16-F10 cells. As shown in Fig. 1C, nanvuranlat inhibited the growth of B16-F10 cells with a GI_50_ value of 23.8 ± 3.6 μM. We also examined the effect of LAT1 knockdown on B16-F10 cell growth. LAT1 stable knockdown cell lines were constructed using shRNA (small-hairpin RNA). The knockdown of LAT1 was confirmed by western blot (Supplementary Fig. S1). LAT1 knockdown cells exhibited reduced cell growth compared to the control (Fig. 1D). Additionally, we investigated the effect of LAT1 inhibition on B16-F10 tumor growth. Nanvuranlat significantly suppressed tumor growth over 14 days of treatment without affecting body weight (Fig. 1E and Supplementary Fig. S2). Furthermore, tumors of B16-F10 with LAT1 knockdown exhibited reduced growth compared to the control (Fig. 1F). These results demonstrate that LAT1 inhibition and LAT1 knockdown suppress B16-F10 cell proliferation and tumor growth (Fig. 1C, D, E and F).

### Inhibition of LAT1 suppresses B16-F10 cell migration

**Figure 2:**
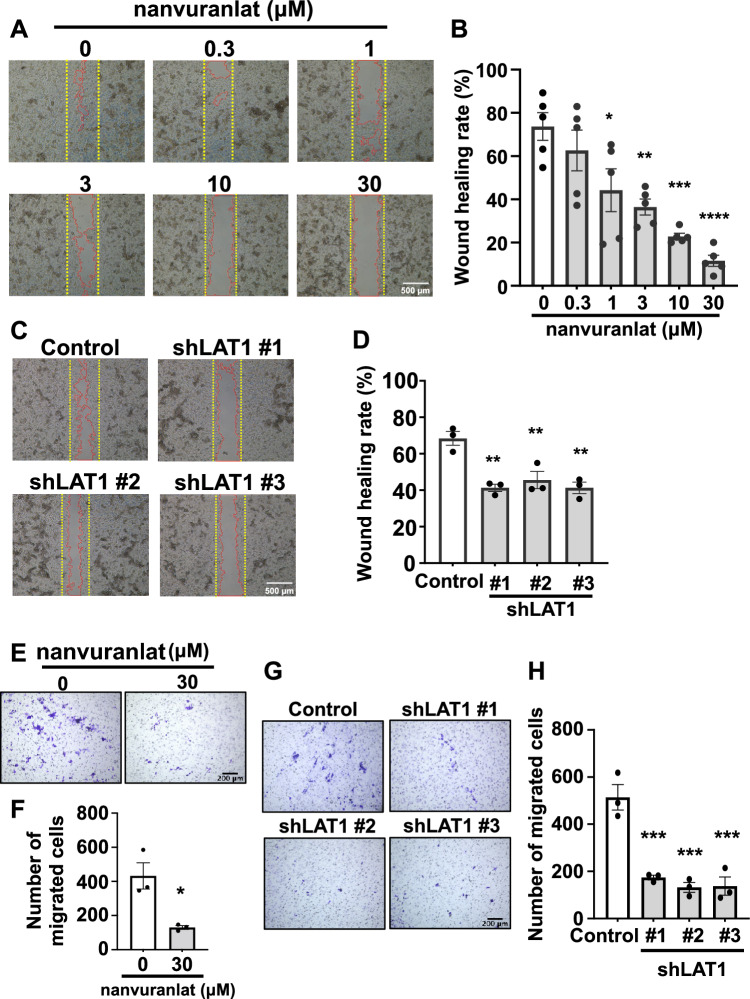
Suppression of cell migration by LAT1 inhibition or knockdown. (**A**) Suppression of B16-F10 cell migration by nanvuranlat. A wound healing assay of B16-F10 cells was performed with the treatment of nanvuranlat (0.3, 1, 3, 10, and 30 μM). Images show representative results of nanvuranlat-treated B16-F10 cells. Cells were allowed to migrate for 8 h (the yellow dotted line represents the initial position of the cell edge (0-h time point), and the cell edges at 8 h are outlined in red). (**B**) Quantification of the wound healing rate (comparison of wound closure between 0-h and 8-h time points) of nanvuranlat-treated B16-F10 cells. (**C**) Suppression of cell migration in B16-F10 LAT1-knockdown cells. Images show the results of B16-F10 shLAT1 cells and control cells (B16-F10 cells transfected with control shRNA). Cells were allowed to migrate for 8 h. (**D**) Quantification of the wound healing rate of B16-F10 LAT1-knockdown cells. (**E**) Suppression of transwell migration by nanvuranlat. A transwell migration assay was performed on B16-F10 cells treated with nanvuranlat (30 μM) for 24 h. The images depict migrated B16-F10 cells following nanvuranlat treatment. (**F**) Quantitative analysis of the number of migrated cells following nanvuranlat treatment in B16-F10 cells. (**G**) Suppression of transwell migration in B16-F10 LAT1-knockdown cells. The images present the migrated cells of both B16-F10 LAT1-knockdown cells and control cells (B16-F10 cells transfected with control shRNA) after a 24-h period of cell migration. (**H**) Quantitative analysis of the number of migrated cells in B16-F10 LAT1-knockdown cells. For (**A**) and (**C**), Scale bars indicate 500 μm. For (**E**) and (**G**), Scale bars indicate 200 μm. For (**B**) and (**D**), the wound healing rate was calculated using the following formula: wound healing rate (%) = [1 − (wound area at 8-h time point/wound area at the 0-time point)] × 100. Statistical significance was determined using a two-tailed unpaired *t*-test (**F**, n = 3) or one-way ANOVA followed by Tukey’s post-test (n = 5 for **B** and n = 3 for **D** and **H**) (* *p* < 0.05, ** *p* < 0.01, *** *p* < 0.001, **** *p* < 0.0001). Data are mean ± SEM.

Using a wound healing assay, we first investigated the effect of LAT1 inhibition on the migration of B16-F10 cells. The rate of cell migration was quantified by comparing the degree of wound closure between the 0-h and 8-h time points. Nanvuranlat decreased cell migration in a concentration-dependent manner compared with the control (Fig. 2A and B). BCH also inhibited B16-F10 cell migration (Supplementary Fig. S3). Additionally, we examined the migration of LAT1-knockdown cells. Compared to the control, LAT1 knockdown cells exhibited a reduction in cell migration (Fig. 2C and D). The inhibitory effect of nanvuranlat or LAT1 knockdown on cell migration was further validated using a transwell migration assay (Fig. 2E–H). These results indicate that inhibition or knockdown of LAT1 decreases the migration of B16-F10 cells.

### Inhibition of LAT1 suppresses B16-F10 cell invasion

**Figure 3:**
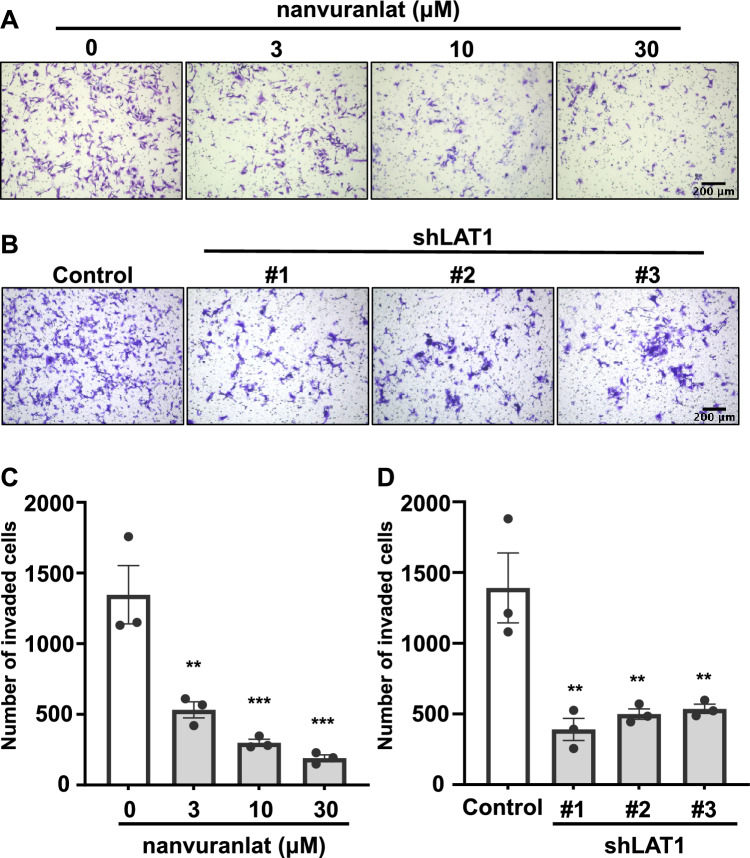
Suppression of cell invasion by LAT1 inhibition or knockdown. (**A**) Suppression of cell invasion by nanvuranlat. A cell invasion assay of B16-F10 cells was performed with the treatment of nanvuranlat (0, 3, 10, and 30 μM) for 24 h. Images show invaded cells of each group. (**B**) Suppression of cell invasion in B16-F10 LAT1-knockdown cells. Images show invaded cells of B16-F10 LAT1-knockdown cells and control cells (B16-F10 cells transfected with control shRNA) after 24 h of cell invasion. Scale bars indicate 200 μm. (**C**) Quantification of the invaded cell number of nanvuranlat-treated B16-F10 cells. (**D**) Quantification of the invaded cell number of B16-F10 LAT1-knockdown cells. Statistical significance was determined using one-way ANOVA followed by Tukey's post-test (n = 3, ** *p* < 0.01, *** *p* < 0.001). Data are mean ± SEM.

We used a Boyden chamber assay to study cell invasion. Both nanvuranlat and BCH significantly decreased the invasion of B16-F10 cells compared with the control (Fig. 3A and B; Supplementary Fig. S3). Furthermore, B16-F10 LAT1-knockdown cells showed a reduction in cell invasion compared to the control (Fig. 3C and D). These results suggest that inhibition of LAT1 can reduce B16-F10 cell invasion.

### Inhibition of LAT1 suppresses the B16-F10 metastasis in the lung metastasis model

**Figure 4:**
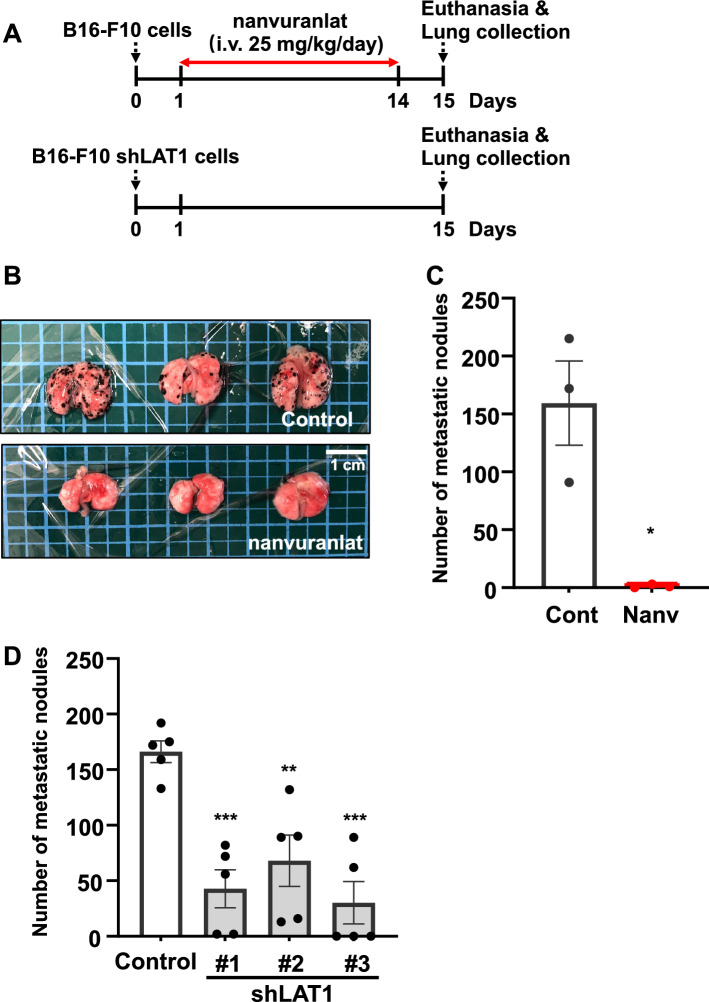
Suppression of lung metastasis by LAT1 inhibition or knockdown in in vivo lung metastasis model. (**A**) Schematic diagram of experimental schedule. Cells were transplanted into mice through intravenous injection. (**B**) Effect of nanvuranlat on B16-F10 lung metastasis. The pictures show the lungs of placebo control mice (upper panel) and mice treated with nanvuranlat (i.v., 25 mg/kg, daily, 14 days, lower panel). (**C**) Quantification of lung metastatic nodules in placebo control and nanvuranlat groups (Cont: placebo control; Nanv: nanvuranlat). (**D**) Quantification of metastatic lung nodules in mice transplanted with control cells (B16-F10 cells transfected with control shRNA) or B16-F10 LAT1-knockdown cells. Statistical significance was determined using a two-tailed unpaired *t*-test (**C**, n = 3) or one-way ANOVA followed by Tukey's post-test (**D**, n = 5) (** *p* < 0.01, *** *p* < 0.001). Data are mean ± SEM.

We transplanted B16-F10 cells or B16-F10 LAT1-knockdown cells through the tail vein of mice. The experimental schedule is depicted in Fig. 4A. Nanvuranlat treatment reduced the formation of metastatic nodules in the lungs (Fig. 4B and C). Similarly, BCH significantly decreased lung metastasis of B16-F10 cells (Supplementary Fig. S4). The number of metastatic lung nodules was reduced in the B16-F10 LAT1-knockdown cell groups compared to the control group (Fig. 4D). These results suggest that the inhibition of LAT1 can decrease lung metastasis of B16-F10 cells in vivo.

### Inhibition of LAT1 suppresses the B16-F10 lung, spleen, and lymph node metastasis in the orthotopic metastasis model

**Figure 5:**
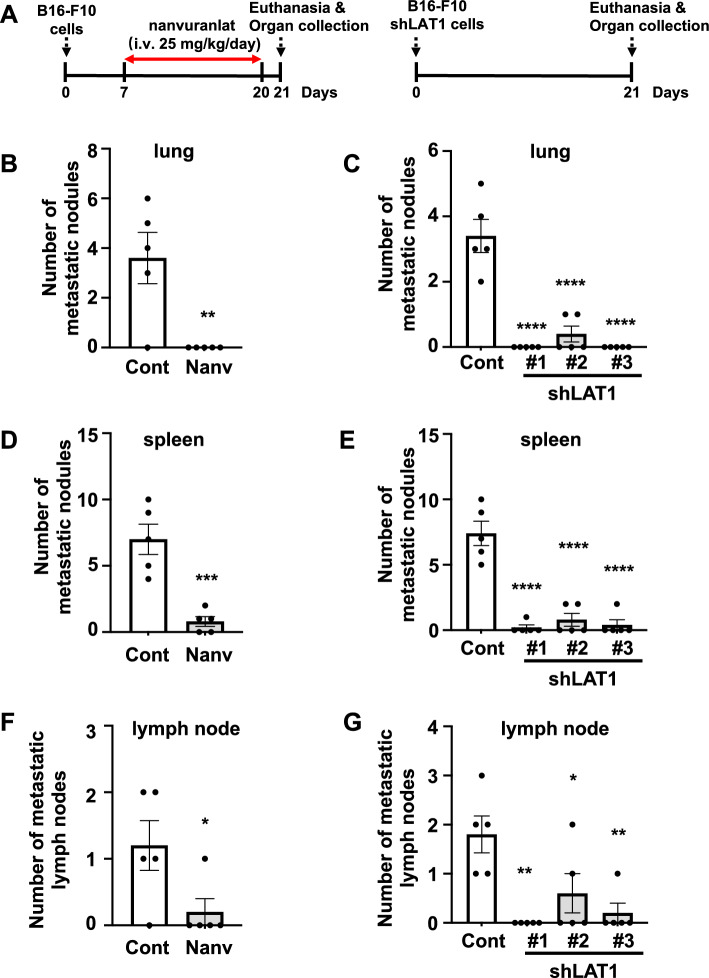
Suppression of metastasis by LAT1 inhibition or knockdown in an orthotopic metastasis model. (**A**) Schematic diagram of the experimental schedule. Cells were transplanted subcutaneously into the hind footpad of mice. (**B**, **D**, **F**) Suppression of B16-F10 metastasis by nanvuranlat. The bar graph shows the quantification of metastatic nodules in the lung (**B**), spleen (**D**), and the number of metastatic lymph nodes (**F**) in placebo control or nanvuranlat treatment (i.v., 25 mg/kg, daily, 14 days) groups (Cont: placebo control; Nanv: nanvuranlat). (**C**, **E**, **G**) Suppression of B16-F10 metastasis by LAT1 knockdown. The bar graph shows the quantification of metastatic nodules in the lung (**C**), spleen (**E**), and the number of metastatic lymph nodes (**G**) in control (Cont, B16-F10 cells transfected with control shRNA) or B16-F10 LAT1-knockdown cell groups. Statistical significance was determined using a two-tailed unpaired *t*-test (**B**, **D**, **F**) or one-way ANOVA followed by Tukey’s post-test (**C**, **E**, **G**) (n = 5, * *p* < 0.05, ** *p* < 0.01, *** *p* < 0.001, **** *p* < 0.0001). Data are mean ± SEM.

B16-F10 cells or B16-F10 LAT1-knockdown cells were transplanted into mouse footpads. The experimental schedule is depicted in Fig. 5A. Compared with the placebo control, the number of metastatic lung nodules was decreased in nanvuranlat-treated mice (Fig. 5B). B16-F10 cell groups with LAT1 knockdown had significantly reduced metastatic lung nodules (Fig. 5C). Similar results were observed in spleen metastasis. Nanvuranlat treatment significantly reduced the formation of B16-F10 spleen metastatic nodules (Fig. 5D). A reduction in spleen metastatic nodules was found in the B16-F10 LAT1-knockdown cell groups, compared to the control (Fig. 5E). Examination of metastatic lymph nodes showed that nanvuranlat treatment and LAT1 knockdown decreased the formation of metastatic lymph nodes (Fig. 5F and G). These data demonstrate that nanvuranlat and LAT1 knockdown decreases lung, spleen, and lymph node metastases of B16-F10 melanoma in the orthotopic metastasis model.

### LAT1 inhibitors downregulate integrin αv and β3 cell surface expression

**Figure 6:**
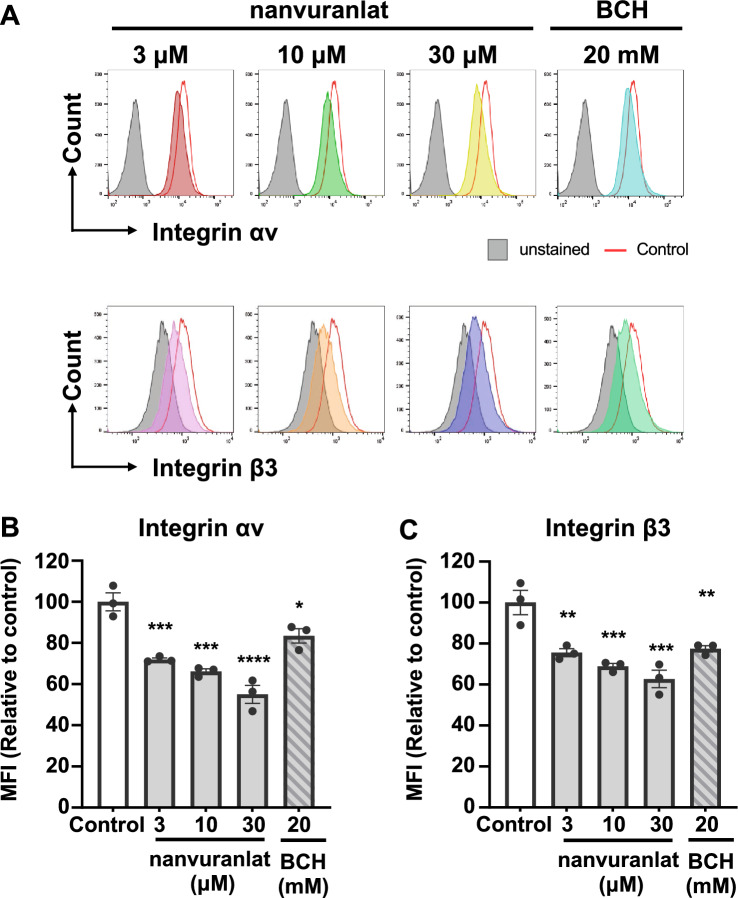
Suppression of integrin αv and β3 cell surface expression by the treatment of LAT1 inhibitors. (**A**) Cell surface expression of integrin αv and integrin β3. Cell surface expression of integrin αv and integrin β3 in B16-F10 cells treated with nanvuranlat (3, 10, and 30 μM) or BCH (20 mM) for 24 h was detected by flow cytometry. Data are represented as histograms showing fluorescence intensity (y-axis) versus cell count (x-axis). Gray-filled peaks represent unstained B16-F10 cells (without inhibitor treatment), red open peaks represent cells without inhibitor treatment, and colored peaks indicate cells treated with the inhibitors. (**B**, **C**) Quantitative analysis of MFI (Median Fluorescence Intensity, relative to cells without inhibitor treatment) of B16-F10 cells treated with nanvuranlat or BCH. Statistical significance was determined using one-way ANOVA followed by Tukey's post-test (n = 3, * *p* < 0.05, ** *p* < 0.01, *** *p* < 0.001, **** p < 0.0001). Data are mean ± SEM.

We used flow cytometry to assess the cell surface expression of integrin αv and integrin β3, which have been implicated in cancer metastasis^26^. After treatment with nanvuranlat or BCH for 24 h, the cell surface expression of integrin αv and integrin β3 was decreased (Fig. 6A). The median fluorescence intensity (MFI, relative to the no-treatment control) of integrin αv and integrin β3 was significantly reduced in the LAT1 inhibitor-treated groups (Fig. 6B and C). These results suggest that LAT1 inhibitors reduce the expression of integrin αv and integrin β3 on the surface of B16-F10 cells.

### LAT1 inhibitors reduce the integrin αv and β3 protein expression and downregulate the mTOR signaling pathway

**Figure 7:**
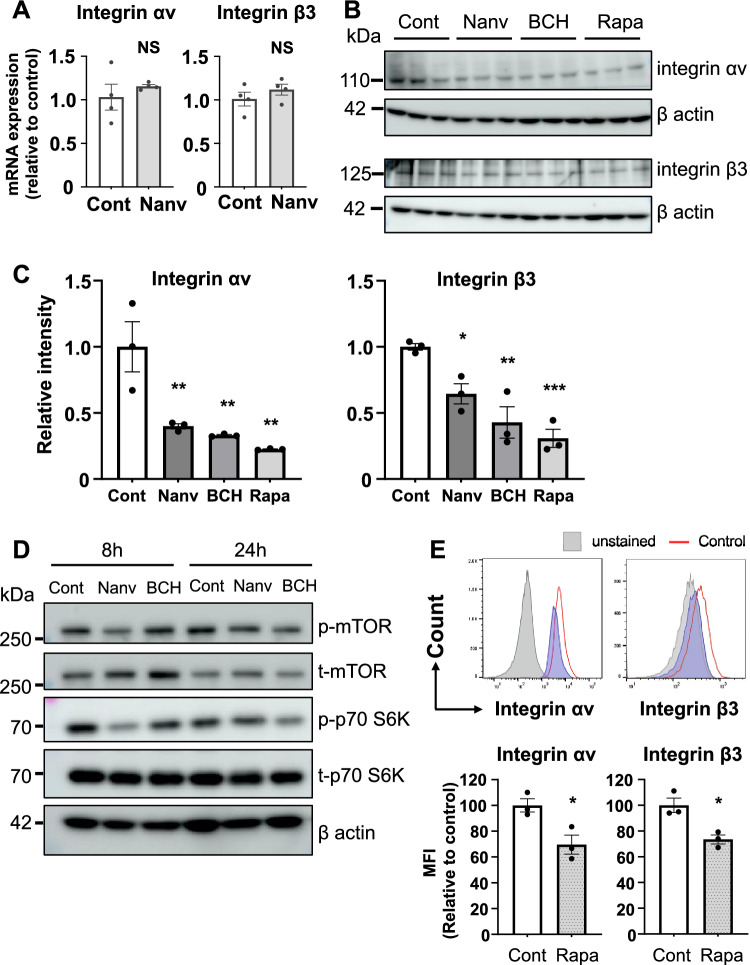
Suppression of protein expression of integrin αv and β3 and downregulation of mTOR signaling pathway by LAT1 inhibitor treatment. (**A**) Quantification of integrin αv and integrin β3 mRNAs after nanvuranlat treatment. B16-F10 cells were treated with 30 μM nanvuranlat for 4 h. Data were normalized to the expression of GAPDH mRNA and are shown as relative expression levels compared to the untreated control. (**B**) Protein expression of integrin αv and integrin β3 with nanvuranlat, BCH, or rapamycin treatment. Protein levels of integrin αv and integrin β3 in B16-F10 cells after 24-h treatment with nanvuranlat (30 μM), BCH (20 mM), or rapamycin (10 nM) were analyzed by western blot. Original, uncropped electrophoretic blots are presented in Supplementary Fig. S5. (**C**) Quantification of the protein levels of integrin αv and integrin β3. (**D**) Effects of nanvuranlat and BCH on mTOR signaling pathway. Protein levels of mTOR (p-mTOR: phospho-mTOR; t-mTOR: total mTOR) and p70 S6K (p-p70 S6K: phospho-p70 S6K; t-p70 S6K: total p70 S6K) in B16-F10 cells after 8- or 24-h treatment with nanvuranlat (30 μM) or BCH (20 mM) were analyzed by western blot. Original, uncropped electrophoretic blots are presented in Supplementary Fig. S5. (**E**) Suppression of cell surface expression of integrin αv and integrin β3 with rapamycin treatment. Upper panel: cell surface expression of integrin αv and integrin β3. Cell surface expression of integrin αv and integrin β3 in B16-F10 cells after 24-h treatment with rapamycin (10 nM) was detected by flow cytometry. Data are represented as histograms showing fluorescence intensity (y-axis) versus cell count (x-axis). Gray-filled peaks represent unstained B16-F10 cells (without rapamycin treatment), red open peaks represent cells without rapamycin treatment, and the blue peak indicates rapamycin-treated cells. Lower panel: quantitative analysis of MFI (relative to control) of B16-F10 cells treated with rapamycin. Abbreviation: Cont: control; Nanv: nanvuranlat; Rapa: rapamycin. Statistical significance was determined using a two-tailed unpaired *t*-test (**A** and **E**) or one-way ANOVA followed by Tukey's post-test (**C**) (n = 3, NS, not significant, * *p* < 0.05, ** *p* < 0.01, *** *p* < 0.001). Data are mean ± SEM.

Downregulation of the mTOR signaling pathway was suggested to reduce the expression of integrin αv and β3^27^. To investigate the potential mechanism underlying the reduction in cell surface expression of integrins αv and β3 following LAT1 inhibition, we examined the mRNA and protein expression of integrins αv and β3 after LAT1 inhibitor treatment. There were no significant differences in the mRNA levels of integrin αv and β3 between the nanvuranlat-treated and control groups (Fig. 7A), suggesting that nanvuranlat may not influence the gene transcription of integrin αv and β3. However, western blot results showed that nanvuranlat and BCH decreased the protein expression of integrin αv and integrin β3 (Fig. 7B). The downregulation of integrin αv and β3 protein levels was verified through western blot band intensity analysis (Fig. 7C). Since LAT1 is involved in the activation of the mTOR signaling pathway^28^, we examined the phosphorylation levels of mTOR and its downstream target p70 S6K in B16-F10 cells treated with LAT1 inhibitors. The results showed that nanvuranlat downregulated the phosphorylation of mTOR and p70 S6K under 8 h of treatment. However, by 24 h, this downregulation was largely restored (Fig. 7D). For BCH, the downregulation of p70 S6K phosphorylation was observed only at 24 h (Fig. 7D). To examine whether the reduction of cell surface integrins was attributed to mTOR downregulation, we checked the effect of rapamycin on cell surface integrin αv and β3 expression. A decrease in integrin αv and β3 on the surface of B16-F10 cells was observed following rapamycin treatment for 24 h (Fig. 7E). Rapamycin also decreased the protein expression level of integrin αv and integrin β3 (Fig. 7C). These results suggest that the decrease in integrin αv and β3 expression caused by LAT1 inhibition may be related to the suppression of mTOR signaling.

## Discussion

In this study, we demonstrated that inhibition of LAT1 by using LAT1 inhibitors (nanvuranlat and BCH) or LAT1 knockdown reduced B16-F10 metastasis. Our in vitro experiments showed that LAT1 inhibition significantly decreased cell migration and invasion. In a lung metastasis mouse model, LAT1 inhibition reduced the formation of B16-F10 lung metastatic nodules. Furthermore, we showed that inhibiting LAT1 led to a reduction in spontaneous lung, spleen, and lymph node metastasis of B16-F10 cells in an orthotopic metastasis model. We also found that the reduced expression of integrin αvβ3 was associated with the decline in B16-F10 metastasis induced by LAT1 inhibition. This study reveals that LAT1 inhibition reduces metastasis, providing new evidence supporting LAT1 as a potential anti-cancer target.

We investigated the effects of LAT1 inhibition on B16-F10 cell migration and invasion in vitro. Our results indicated that both LAT1 inhibitors (nanvuranlat and BCH) and LAT1 knockdown significantly reduced B16-F10 cell migration and invasion (Figs. 2, 3, and Supplementary Fig. S2). Given that the doubling time of B16-F10 cells has been reported to be approximately 20.1 h^29^, the cell count in the 8-h observation period for the wound healing assay is expected to increase by only 1.3 times. In the case of the invasion assay, which was conducted under starvation conditions (without FBS), cell growth is anticipated to decelerate, thereby extending the doubling time. This study's observation period for cell invasion was 24 h, roughly equivalent to the reported doubling time of B16-F10 cells under regular serum-containing conditions. Therefore, while the inhibition of LAT1 can suppress cell growth, the impact of this growth inhibition on cell migration and invasion is projected to be minimal within our experimental conditions. Cell migration requires the coordination of cytoskeletal dynamics and reorganization^30^. We previously showed that nanvuranlat increases the phosphorylation levels of Rho-associated protein kinase (ROCK), myosin light-chain kinase (MLCK), focal adhesion kinase (FAK), and paxillin^31^. Among these, increased phosphorylation of ROCK and MLCK leads to increased non-muscle myosin II phosphorylation, which results in large actin bundles and stable adhesions, thereby inhibiting cell migration^32^. Impaired FAK and paxillin signaling increases cell migration, so when nanvuranlat upregulates the phosphorylation levels of FAK and paxillin, it, in turn, negatively regulates cell migration^33^. On the other hand, previous research has indicated that inhibiting FAK suppresses cell migration in esophageal adenocarcinoma cell lines^34^. The divergent roles of FAK in modulating cell motility could be attributed to variations in cell systems and phosphorylation sites^35^. We speculate that LAT1 inhibition suppressed cell migration, at least in part, by modulating these signaling pathways governing cytoskeletal dynamics.

Cell migration is a prerequisite for invasion, which requires cells to adjust their shape and interact with the extracellular matrix (ECM) to move through the matrix^36^. The mitogen-activated protein kinase (MAPK) plays a crucial role in promoting cell invasion by inducing the expression of proteolytic enzymes that degrade the basement membrane^37^. Nanvuranlat has been shown to inhibit the phosphorylation of MAPK^38^, suggesting that nanvuranlat could regulate cell invasion through MAPK. Therefore, we propose that LAT1 inhibition suppresses cell migration and invasion by modulating the key signaling pathways which govern cytoskeletal dynamics and interactions with ECM.

In this study, we demonstrated that LAT1 inhibition reduced cancer metastasis in animal models. Using the B16-F10 lung metastasis model, we showed that LAT1 inhibition significantly reduced the formation of metastatic nodules in the lung (Fig. 4). Moreover, in the orthotopic metastasis model, LAT1 inhibition reduced sentinel lymph node metastasis and significantly decreased distant lung and spleen metastases (Fig. 5B–G). At the primary site of cancer, enhanced angiogenesis is associated with cancer cell intravasation, as increased tumor microvessel density raises the potential for tumor cells to enter the bloodstream^39^. LAT1 has been implicated in tumor angiogenesis^17^. Inhibition of LAT1 using nanvuranlat interferes with VEGF-A/VEGFR2 and mTOR signaling, thereby participating in the regulation of angiogenesis^17^. Therefore, it is suggested that the suppression of angiogenesis by LAT1 inhibition could contribute to the reduction in metastasis. Additionally, nanvuranlat treatment has been shown to induce G1 arrest by regulating the expression of cell cycle-related proteins and phosphorylation of cell cycle-related kinases^31^. We speculate that nanvuranlat-induced cell cycle arrest, which inhibits tumor growth at both primary and metastatic sites, could also contribute to the apparent suppression of metastasis. Collectively, LAT1 inhibition may exert a multifaceted influence on cancer metastasis progression by simultaneously affecting multiple aspects.

Integrins are a family of transmembrane heterodimer proteins composed of various combinations of 18 α and 8 β integrin subunits^40^. Among the integrin family, β3 integrin expression is elevated in cell lines with high metastatic ability compared to those with low metastatic ability^41, 42^. It is primarily associated with the ability of tumors to metastasize ^43^. Integrin β3 forms heterodimers with αIIb and αv subunits, with integrin αvβ3, rather than αIIbβ3, strongly supporting metastasis in melanoma cells^44^. Integrin αvβ3 expression levels correlate with the metastatic propensity in melanoma, breast, lung, prostate, pancreatic, and renal cancer^45–49^. Integrin αvβ3 plays diverse roles in different stages of the metastatic process^26^. For example, integrin αvβ3 binds to matrix metalloproteinase 2 (MMP-2) and serves as a receptor for surface-localized metalloproteinase activity, while MMP-2 binds poorly to other integrins such as αvβ5 and α5β1^50^. Cooperation between αvβ3 integrins and MMP-9 has also been observed^51^. MMP-2 and MMP-9 facilitate cell-mediated ECM degradation, directly regulating cell invasion^52^. Additionally, integrin αvβ3 is implicated in regulating angiogenesis among all integrins^53^. Collectively, integrin αvβ3 may be one of the integrins most closely associated with cancer metastasis. Therefore, we examined changes in the expression of integrin αv and β3 in response to LAT1 inhibition. Our flow cytometry results indicated that cell surface expression of integrin αv and β3 was reduced by LAT1 inhibition (Fig. 6B and C), suggesting that integrin αv and β3 are involved in the decline of cancer metastasis induced by LAT1 inhibition.

Interestingly, previous studies have indicated that rapamycin, an mTOR inhibitor, decreased the expression of integrin αv^27^. However, the mechanism underlying the effect of mTOR on integrin αv was not elaborated in that study^27^. Our western blot results showed that protein expression levels of integrin αv and β3 decreased under LAT1 inhibitor treatment (Fig. 7B). mRNA results indicated that LAT1 inhibitor nanvuranlat had little effect on integrin αv and β3 gene expression (Fig. 7A). Since integrin αv and β3 did not change at the gene transcription level, regulation may occur at the protein level. We confirmed that inhibiting LAT1 using nanvuranlat or BCH downregulated the mTOR signaling pathway (Fig. 7C). mTOR is an essential regulator of protein synthesis^54^. It has been demonstrated that nanvuranlat suppresses protein synthesis by influencing nutrient-sensing mTORC1 and GAAC pathways^55^. Meanwhile, mTOR is a key regulator of autophagy^56^. Stimulation of autophagy increased the co-localization of integrin with autophagic vacuoles and their subsequent degradation by lysosomes as the autophagic pathway merged with the endocytic pathway^57, 58^. Additionally, it has been shown that inhibition of mTOR promotes ligand-engaged integrin internalization and degradation in lysosomes^59, 60^. Considering that the half-life of integrins on the cell surface is 12–24 h^61^, enhanced internalization and lysosomal degradation of integrins due to downregulated mTOR could significantly reduce cell surface integrins within approximately 24 h. Thus, the decrease in integrin αv and β3 cell surface expression induced by 24-h LAT1 inhibitor treatment could be related to mTOR downregulation. Moreover, we also confirmed that mTOR downregulation reduced cell surface expression of integrin αv and β3 using rapamycin (Fig. 7E). Therefore, we speculate that mTOR downregulation induced by LAT1 inhibition decreased the protein expression level of integrin αv and β3 by (1) interfering with integrin protein synthesis; and (2) enhancing internalization and lysosomal degradation of integrin on the cell surface, thereby suppressing metastasis.

To date, anti-metastasis targets have mainly focused on affecting cell migration and invasion capacity^62^. Based on their molecular mechanisms, anti-metastasis agents can be categorized as those involving the cytoskeleton or ECM remodeling^62^. Y-27632, a selective ROCK inhibitor, has decreased cell invasion in various cancer cell types^62^ and reduced liver metastasis of HT-29 colorectal cancer in vivo^63^. However, in U87 glioma cells and OCUM-2MD3 gastric carcinoma cells, Y-27632 exhibited the opposite effect, increasing cell invasion^64^. These studies reveal that the contribution of ROCK inhibitors to cancer cell migration and invasion varies among cell lines^64^. MMP inhibitors (MMPIs) were developed as anti-metastasis agents due to their ability to inhibit cancer cell invasion^65^. However, their lack of specificity and selectivity may cause serious adverse effects, which could explain why MMPIs have failed to demonstrate satisfactory efficacy in clinical trials^66^. For instance, marimastat, a broad-spectrum MMPI, did not improve survival in patients with pancreatic cancer^67^. Other potential anti-metastasis agents with promising preclinical efficacy that failed to show effects in clinical trials include cilengitide, an RGD peptide antagonist targeting integrin αvβ3 and αvβ5^65^. The lack of cancer specificity poses a significant challenge in developing anti-metastasis agents. Their impact on normal cell function can lead to serious adverse effects poorly tolerated by patients^65^. Given the cancer cell specificity of LAT1 and its ability to reduce metastasis, we propose that LAT1 inhibition could suppress primary cancer growth and potentially reduce cancer metastasis.

In summary, we have revealed an anti-metastasis effect of LAT1 inhibition. Our results demonstrated that the LAT1 inhibitor nanvuranlat reduced metastatic nodule formation in metastasis mouse models, illustrating the potential of using nanvuranlat to decrease or prevent cancer metastasis. Therapeutic strategies targeting LAT1 could be applied to anti-cancer treatment, ultimately improving the prognosis for cancer patients.

## Materials and methods

### Cell culture

Mouse melanoma B16-F10 cells (ATCC^®^CRL-6475™) were grown in RPMI-1640 (Nacalai Tesque, Kyoto, Japan) supplemented 10% FBS (Nichirei, Tokyo, Japan), 100 units/mL penicillin, and 100 μg/mL streptomycin (Nacalai Tesque) at 37 °C with 5% CO_2_, and 95% humidity.

### Chemicals

Nanvuranlat ((2S)-2-amino-3-[4-[(5-amino-2-phenyl-1,3-benzoxazol-7-yl)methoxy]-3,5-dichlorophenyl]propanoic acid, CAS No.: 1037592-40-7) (2HCl salt; purity > 99%), nanvuranlat-sulfobutylether-β-cyclodextrin (nanvuranlat-SBECD), and sulfobutylether-β-cyclodextrin (SBECD, placebo) were provided by J-Pharma Co., Ltd (Kanagawa, Japan)^17^. 2-Aminobicyclo[2.2.1]heptane-2-carboxylic acid (BCH) was purchased from Sigma-Aldrich (St. Louis, MO, USA).

### Quantitative RT-qPCR

Quantitative real-time PCR was performed as described previously^68^. Total RNA was extracted using Isogen II (Nippon Gene, Tokyo, Japan), and reverse transcription was performed for cDNA synthesis (Primescript RT Master Mix, Takara Bio, Shiga, Japan). Quantitative PCR was performed with SYBR Premix ExTaq™ (Tli RNaseH Plus,  Takara Bio) using the 7900HT Fast Real-Time PCR system. The following primers were used: LAT1 (forward: 5′-cgggctgcctgtctacttc- 3′, reverse: 5′-cagagcaccgtcacagagaa-3′), LAT2 (forward: 5′- tggaagaagcctgacattcc- 3′, reverse: 5′- gcccagaacagcaggtagat-3′), LAT3 (forward: 5′- tctctcatcagtgccgtgtt- 3′, reverse: 5′- agtaggaggcccaggttcac-3′), LAT4 (forward: 5′- tgtgggttttggggtgac- 3′, reverse: 5′- tgcaggacgaaggagaagat-3′), 4F2hc (forward: 5′-atggtgcagctggagtgtg-3′, reverse: 5′-ccccgtagctaaaaacagga-3′), integrin αv (forward: 5′-ggtgtggatcgagctgtctt-3′, reverse: 5′- caaggccagcatttacagtg-3′), integrin β3 (forward: 5′-cgggctaaccgaccaggtgtcccg-3′, reverse: 5′- cctgcatgatggcgtcaaag-3′), and GAPDH (forward: 5′-tgcccccatgtttgtgatg-3′, reverse: 5′- tgtggtcatgagcccttcc-3′). The obtained data were normalized to the expression of GAPDH mRNA.

### shRNA-mediated gene knockdown

pLKO.1 lentiviral vector expressing mouse LAT1 shRNA#1 (3359slc1, 5′-ccggcctgagatagtgctgtggttactcgagtaaccacagcactatctcaggtttttg-3′), #2 (988slc1,5′-ccggcctatttcctaccctctctactcgagtagagagggtagtgaaataggtttttg-3′), #3 (233slc1,5′-ccgggcgcaatatcacgctgctcaactcgagttgagcagcgtgatattgcgctttttg-3′) as well as control shRNA (SHC002, 5′-ccggcaacaagatgaagagcaccaactcgagttggtgctcttcatcttgttgttttt-3′) were purchased from Sigma Aldrich.

The shRNA plasmids were used to produce lentiviral particles with the Lentiviral High Titer Packaging Mix (Takara Bio). The resulting lentiviruses were collected from the supernatant of HEK 293 T cells and used to transduce the target cells. B16-F10 cells were seeded in 6-well plates at a density of 2 × 10^5^ cells/well and transduced with the shRNA lentiviral vectors. Two days post-transduction, the cells were selected using puromycin (InvivoGen, San Diego, CA, USA). The knockdown efficiency was confirmed via western blot analysis.

### Western blot analysis

Protein sample preparation and western blot analysis were performed as described previously^69, 70^. Crude membrane samples were prepared for the detection of LAT1 and 4F2hc expression. Cells were collected in lysis buffer (50 mM Tris–HCl (pH 7.4), 150 mM NaCl, 1 mM EDTA, 1 mM phenylmethylsulfonyl fluoride (PMSF), and 10% glycerol) supplemented with 1× protease inhibitor cocktail (Roche Applied Science, Indianapolis, IN, USA). The lysates were solubilized in 1% NP-40 for 30 min before mixing with 5× SDS loading buffer (250 mM Tris–HCl (pH 7.4), 10% SDS, 30% glycerol, 0.05% Bromophenol blue, 0.5 M DTT). Total protein samples were prepared to detect mTOR signaling pathway components and integrin αv and β3. Cells were collected in RIPA lysis buffer (50 mM Tris–HCl (pH 7.4), 150 mM NaCl, 1% NP-40, 0.5% Sodium deoxycholate, 1% SDS, and 1 mM phenylmethylsulfonyl fluoride) supplemented with 1× protease inhibitor cocktail and 1× phosphatase inhibitor cocktail (Roche). After mixing with 5× SDS loading buffer, the lysates were subjected to SDS–polyacrylamide gel electrophoresis (SDS-PAGE) and transferred to PVDF membranes. Detection was performed using ECL Western blotting substrate (Bio-Rad, Hercules, CA, USA), and the signals were visualized with an Amersham Imager 680 (GE Healthcare). The western blot band intensities were measured with ImageJ software (NIH, USA). Integrin αv and integrin β3 protein levels were normalized to β actin levels. The primary antibodies used were as follows: anti-LAT1 (TransGenic, KE026, 1:1000), anti-4F2hc (Santa Cruz Biotechnology, sc-31251, 1:500), anti-integrin β3 (Santa Cruz Biotechnology, sc-14009, 1:200), anti-integrin αv (Santa Cruz Biotechnology, sc-376156, 1:200), anti-β actin (Proteintech, Chicago, IL, USA, 66009-1-Ig, 1:1000), anti-mTOR(7C10) (#2983, 1:500), anti-phospho-mTOR(Ser2448) (#5536, 1:500), anti-p70 S6 Kinase (#9202, 1:500), and anti-phospho-p70 S6 Kinase(Thr389)(108D2)(#9234,1:500). Antibodies against total and phosphorylated mTOR and p70S6 Kinase were purchased from Cell Signaling Technology (Danvers, MA).

### Cell proliferation assay

To determine the 50% growth inhibition (GI_50_) of nanvuranlat, a cell proliferation assay was performed using the Cell Counting Kit-8 (CCK-8, Dojindo, Kumamoto, Japan). B16-F10 cells were seeded in 96-well plates at a density of 1000 cells/well and cultured for 24 h, at which point the time was designated as 0 h. Cells were then treated with varying concentrations of nanvuranlat (1.5, 3, 7, 15, and 30 μM) for 48 h. Following nanvuranlat treatment, cell proliferation was assessed using the CCK-8 assay. Proliferation inhibition was calculated using the following equation: proliferation inhibition (%) = (C − T)/(C − T_0_) × 100, where C represents the cell number at 48 h for the control, T represents the cell number at 48 h for each nanvuranlat concentration, and T_0_ represents the cell number at 0 h. The GI_50_ value was determined through nonlinear regression analysis using GraphPad Prism 9. For the LAT1 knockdown experiment, the proliferation of B16-F10 shLAT1 cells was measured at 24, 48, and 72 h after cell seeding.

### B16-F10 tumor model

Animal experiments were conducted following the protocol and methods approved by the Animal Care Ethics Committee of Osaka University Graduate School of Medicine and Graduate School of Science. All methods were in accordance with the guidelines and regulations established by Osaka University. This study was conducted in compliance with the Animal Research: Reporting of In Vivo Experiments (ARRIVE) guidelines. Six-week-old female C57BL/6 mice were purchased from Japan SLC, Inc. (Shizuoka, Japan). B16-F10 cells were subcutaneously transplanted into the footpad of the hind leg (2 × 10^5^ cells per mouse). After 7 days, the mice were randomly assigned to groups and began daily nanvuranlat-SBECD (25 mg/kg) or placebo administration (i.v.) for 14 days. Body weight and tumor size were recorded at a fixed time each day before nanvuranlat-SBECD administration. Mice were sacrificed the day following the completion of nanvuranlat treatment (Day 21). Mice transplanted with B16-F10 LAT1-knockdown cells or control cells (B16-F10 cells transfected with control shRNA) were also sacrificed on Day 21. Tumor size was measured with a caliper, and tumor volume was calculated using the formula: (length × width^2^)/2 (mm^3^).

### Wound healing assay

The wound healing assay was performed as previously described^17^. Briefly, cells were seeded in Culture-Insert 2 Well (ibidi, Martinsried, Germany) at 1 × 10^5^ cells per 100 μL per well, placed in a 24-well plate. After being cultured overnight, the insert was removed, and 500 μL of RPMI-1640 medium containing nanvuranlat (0.3, 1, 3, 10, and 30 μM) or BCH (3, 10, and 30 mM) was added to the well. LAT1-knockdown and control wells received RPMI-1640 medium only. The cell-free edges were photographed to record the initial position of cell migration immediately after the removal of inserts (0-h time point). Cells were allowed to migrate for 8 h. The cell migration area at 0-h and 8-h time point was measured using the wound healing plugin of ImageJ software. The wound healing rate was calculated using the following formula: wound healing rate (%) = [1 − (wound area at the 8-h time point/wound area at the 0-h time point)] × 100.

### Transwell migration assay

Chambers featuring a 6.5 mm diameter and an 8-μm pore polycarbonate membrane (Kurabo, Osaka, Japan) were utilized to examine the migration of B16-F10 cells. B16-F10 cells were suspended in 500 μL of FBS-free RPMI-1640 medium and seeded into the upper chamber at a density of 2.5 × 10^5^ cells per well. The lower chamber was filled with RPMI-1640 medium supplemented with 10% FBS. In the case of LAT1 inhibitor treatment, 30 μM of nanvuranlat was added to both chambers. Following 24 h of cell migration, cells that had migrated were fixed using 4% paraformaldehyde (PFA) in phosphate-buffered saline (PBS, pH 7.4) and stained with 0.5% crystal violet. The number of migrated cells was determined visually according to images captured from the bottom of the chamber using a bright-field microscope (BE-9000, Keyence, Osaka, Japan).

### Cell invasion assay

Chambers (6.5 mm in diameter with 8-μm pores polycarbonate membrane, Kurabo, Osaka, Japan) coated with Matrigel (Corning, NY, USA) were used to study the cell invasion of B16-F10 cells. B16-F10 cells suspended in 500 μL FBS-free RPMI-1640 medium were seeded in the upper chamber at a density of 5 × 10^5^ cells. The lower chamber was filled with RPMI-1640 medium containing 10% FBS. For LAT1 inhibitor treatment, nanvuranlat (3, 10, and 30 μM) or BCH (10 and 30 mM) was added to both sides of the chamber. After 24 h of cell invasion, invaded cells were fixed in 4% paraformaldehyde (PFA) in phosphate-buffered saline (PBS, pH 7.4) and stained with 0.5% crystal violet. The number of invaded cells was counted by the naked eye according to the pictures taken from the bottom of the chamber using a bright-field microscope (BE-9000, Keyence, Osaka, Japan).

### Lung metastasis mouse model

On Day 0, B16-F10 cells were transplanted into C57BL/6 mice through intravenous injection (2 × 10^5^ cells per mouse). After transplantation, mice were randomly divided into a control group and a treated group. Nanvuranlat-SBECD (25 mg/kg/day), BCH (200 or 400 mg/kg/day), or an equivalent amount of placebo in saline was intravenously administered (i.v.) once daily for 14 days. Mice were sacrificed on Day 15, and the lungs were excised. Mice transplanted with B16-F10 LAT1 knockdown cells or control cells (B16-F10 cells transfected with control shRNA) were also sacrificed on Day 15. Colonies with a diameter greater than 0.5 mm were identified as metastatic nodule. The number of metastatic nodules on the surface of each lung lobe was counted by the naked eye.

### Orthotopic metastasis model

On Day 0, B16-F10 cells were transplanted subcutaneously into the footpad of the hind leg (2 × 10^5^ cells per mouse). After 7 days, the mice were randomly grouped and started on nanvuranlat-SBECD (25 mg/kg/day) or placebo administration (i.v.) for 14 days. Mice were sacrificed on Day 21, followed by the removal of the lungs and spleens. Mice transplanted with B16-F10 LAT1 knockdown cells or control cells (B16-F10 cells transfected with control shRNA) were also sacrificed on Day 21. The number of metastatic nodules on the surface of the lungs and spleens was counted. B16-F10 metastases in the sentinel lymph nodes (popliteal and inguinal lymph nodes) were examined. Metastatic lymph nodes were identified as those with visible melanin (black pigment).

### Flow cytometry experiment

B16-F10 cells were seeded in 24-well plates at 1 × 10^5^ cells/well, allowed to grow for 24 h, and then treated with nanvuranlat (3, 10, and 30 μM), BCH (20 mM), or rapamycin (10 nM) for another 24 h. After that, cells were washed twice with PBS, detached by trypsin (Nacalai Tesque), resuspended in MACS buffer (0.5% BSA and 2 mM EDTA in PBS), and kept on ice for antibody incubation. Cells (1 × 10^6^ cells) were incubated for 30 min on ice with fluorescein isothiocyanate (FITC)- and phycoerythrin (PE)-conjugated monoclonal antibodies directed against the following integrin subtypes: anti-αv (104105, BioLegend, San Diego, USA) and β3 (104305, BioLegend). After washing with MACS buffer three times, cells were suspended in 500 μL of MACS buffer for analysis. Unstained B16-F10 cells were used as the negative control, and no inhibitor-treated B16-F10 cells were used as a control for inhibitor-treated cells. Integrin cell surface expression of B16-F10 cells was measured using an Attune Flow Cytometer (Thermo Fisher Scientific, Waltham, MA, USA) and analyzed using FlowJo software (Becton, Dickinson and Company, Franklin Lakes, NJ, USA). The median fluorescence intensity (MFI) relative to the control was generated from three independent replicate trials.

### Statistical analysis

The statistical analyses were performed using unpaired two-tailed Student's *t*-test or one-way or two-way ANOVA followed by Tukey’s post-test or Sidak's post-test with GraphPad Prism 9 (GraphPad Software Inc., San Diego, CA, USA). Data are presented as mean ± SE.M. For all analyses, *p*-values less than 0.05 were considered statistically significant.

### Supplementary Information

Supplementary Figures.

## Data Availability

All data generated or analyzed during this study can be found in the published article and Supplementary Information files.
