# Supplementary material for: Inhibition of cancer-type amino acid transporter LAT1 suppresses B16-F10 melanoma metastasis in mouse models
Source: Sci Rep. 2023 Aug 25;13:13943. doi: 10.1038/s41598-023-41096-3 (PMC10457391; doi:10.1038/s41598-023-41096-3)
Supplement: Supplementary file 1 — Supplementary Figures. [file 41598_2023_41096_MOESM1_ESM.pdf]

Supplementary Figure S1

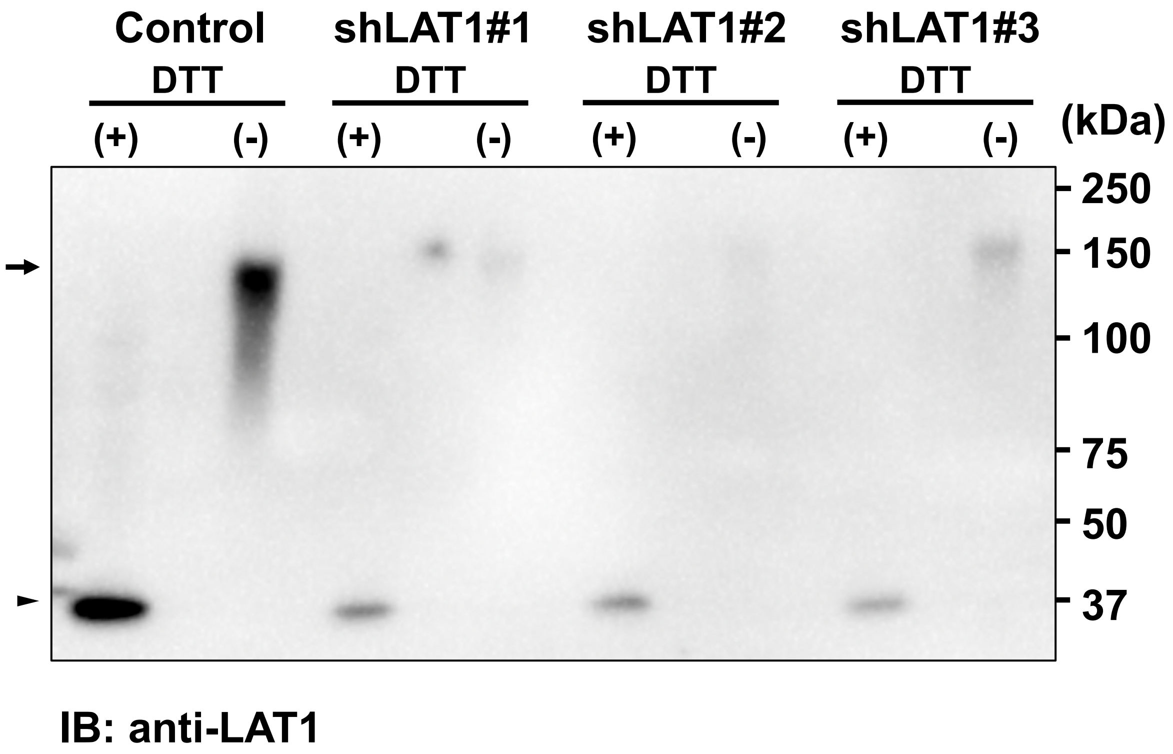

Supplementary Figure S1. Confirmation of LAT1 knockdown in B16-F10 cells. Western blot analysis of LAT1-knockdown B16-F10 cells. Expression of LAT1 in B16-F10 cells transfected with control shRNA or LAT1 shRNA (shLAT1 #1, #2, and #3) was detected using an anti-LAT1 antibody under reducing (DTT(+))and non-reducing (DTT(-))) conditions. Arrows indicate the bands corresponding to the LAT1-4F2hc heterodimer at 150 kDa. Black arrowheads indicate the bands of LAT1 monomers (37 kDa).

Supplementary Figure S2

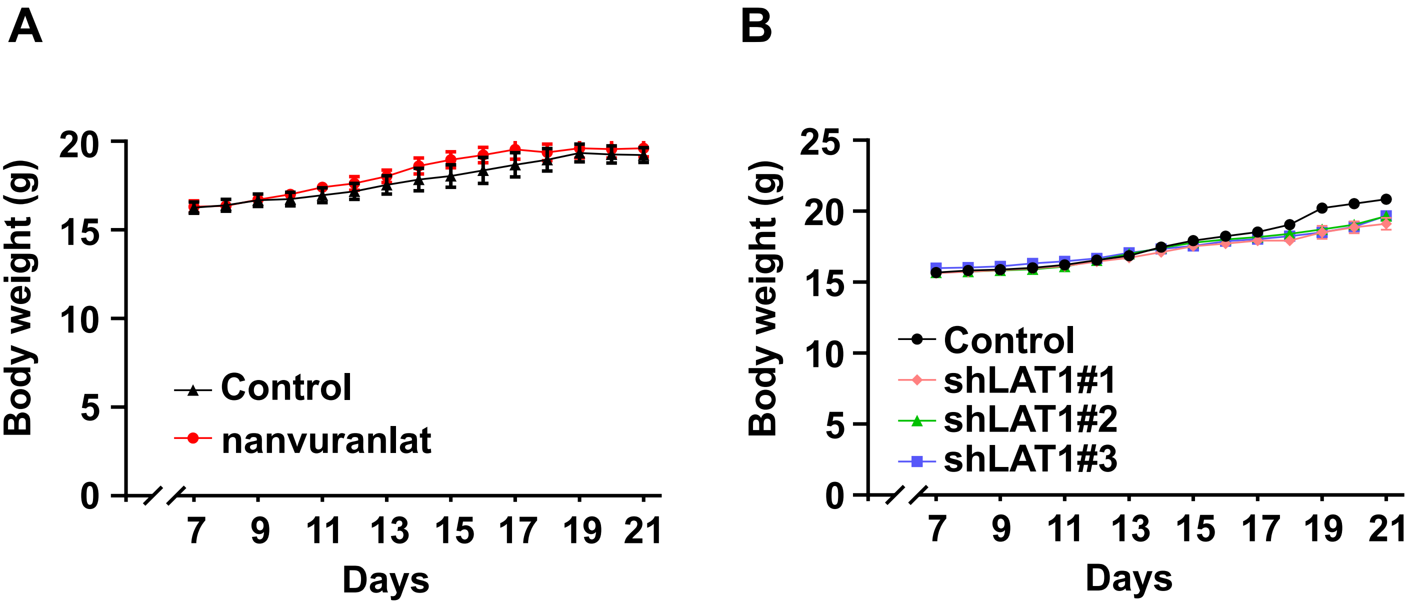

Supplementary Figure S2. Body weight of B16-F10 tumor-bearing mice. ***A*****.** Body weight of mice during two weeks of nanvuranlat administration. ***B*****.** Body weight of mice during a two-week observation period in each B16-F10 shLAT1 cell group.

Supplementary Figure S3

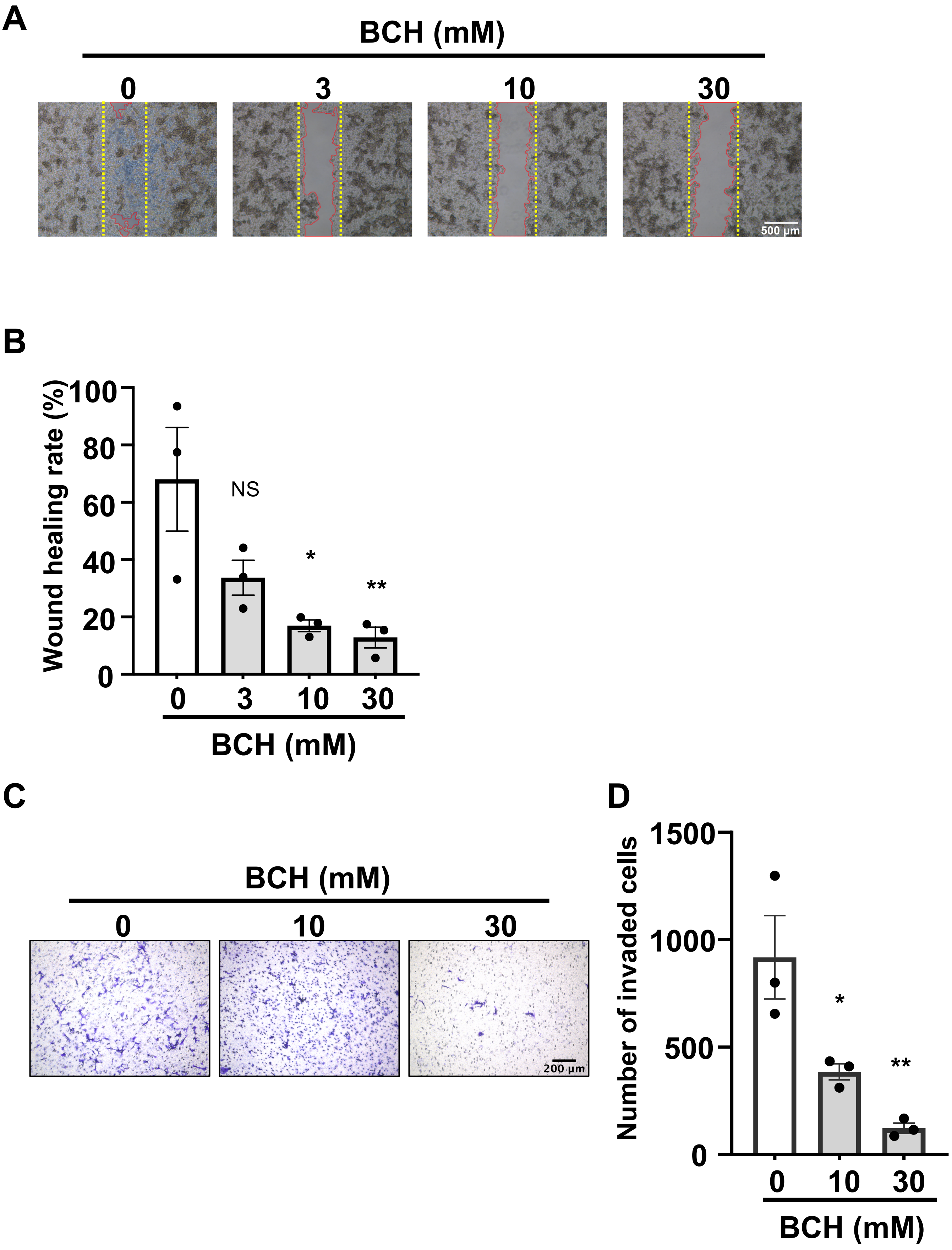

Supplementary Figure S3. Suppression of cell migration and invasion by BCH. ***A.*** Suppression of B16-F10 cell migration by BCH. A wound healing assay of B16-F10 cells was performed with BCH treatment (3, 10, and 30 mM). Images display representative results of BCH-treated B16-F10 cells. Cells were allowed to migrate for 8 hours (the yellow dotted line represents the initial position of the cell edge, and the cell edges at 8 hours are outlined in red). ***B.*** Quantification of the wound healing rate of BCH-treated B16-F10 cells. ***C.*** Suppression of cell invasion by BCH. Images show invaded B16-F10 cells after 24 hours of BCH treatment. ***D***. Quantification of the number of invaded cells in BCH-treated B16-F10 cells.

Statistical significance was determined using one-way ANOVA followed by Tukey's post-test (n=3, NS, not significant, * $p < 0.05$, ** $p < 0.01$). Data are presented as mean ± SEM. Scale bars: A, 500 µm; C, 200 µm.

Supplementary Figure S4

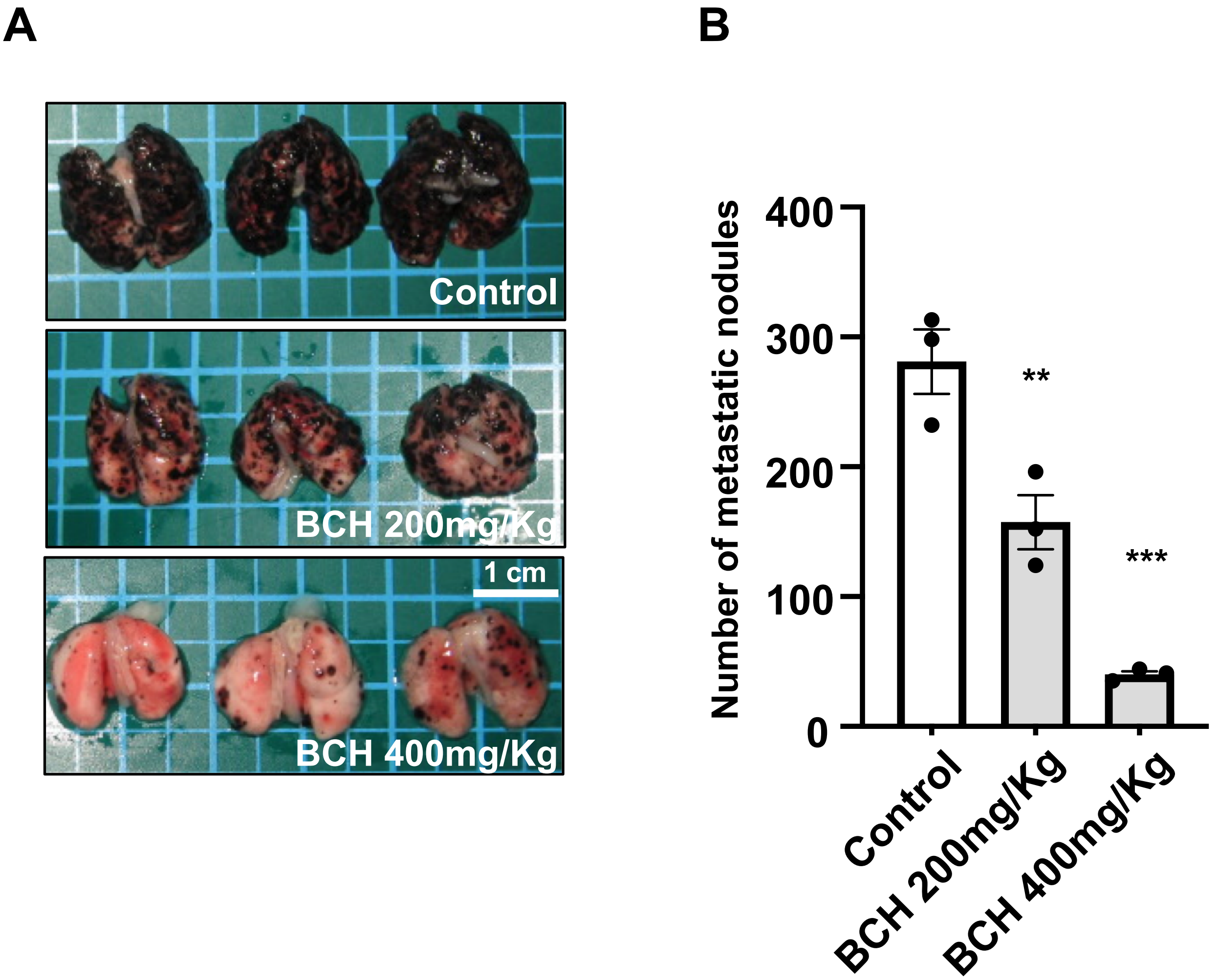

Supplementary Figure S4. Inhibition of B16-F10 lung metastasis by BCH in *in vivo* lung metastasis model. ***A.*** BCH suppressed B16-F10 lung metastasis compared to the control. Images display the lungs of control (saline) and BCH-treated mice (*i.v.*, 200 or 400 mg/kg, daily, 14 days). ***B.*** Quantification of metastatic nodules in control (saline) or BCH treatment groups.

Statistical significance was determined using one-way ANOVA followed by Tukey's post-test (n=3, ** $p < 0.01$, *** $p < 0.001$). Data are presented as mean ± SEM.

Supplementary Figure S5

A

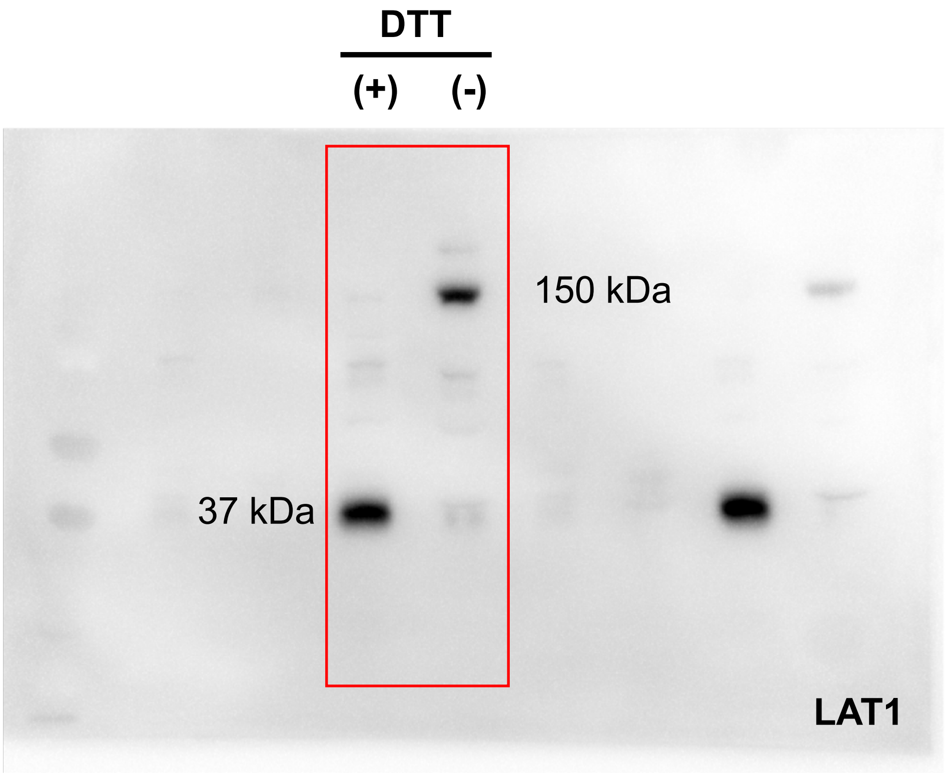

B

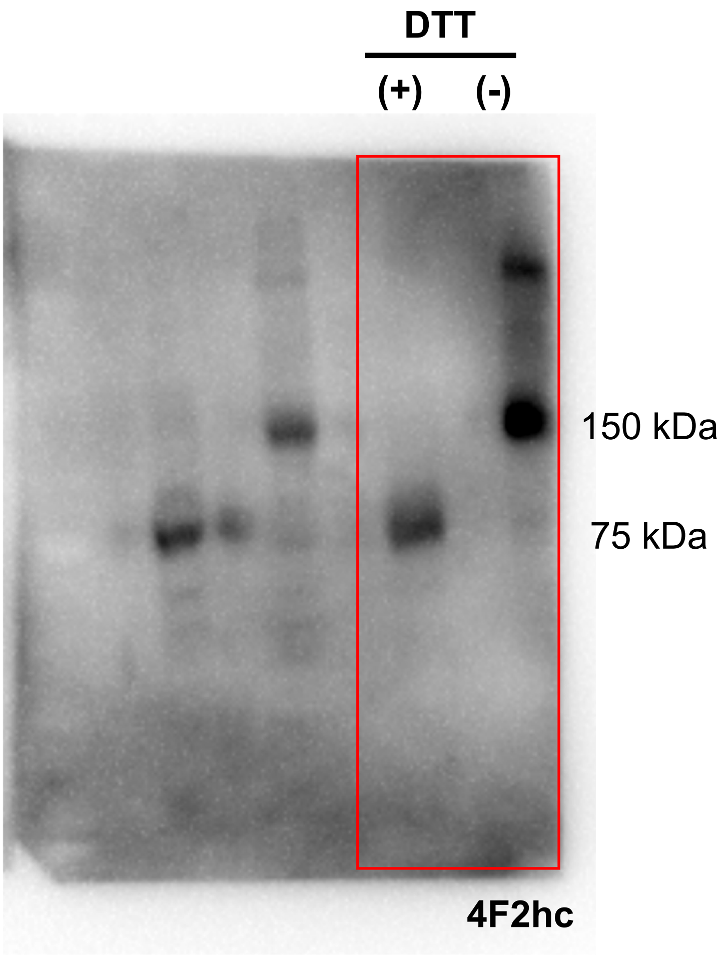

C

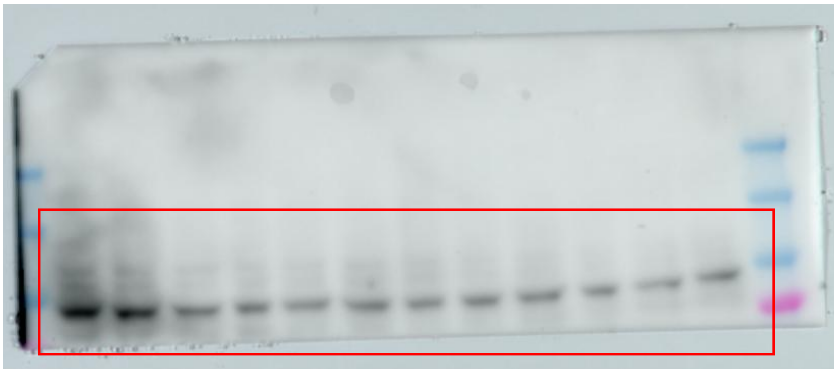
110 kDa
integrin αv

D

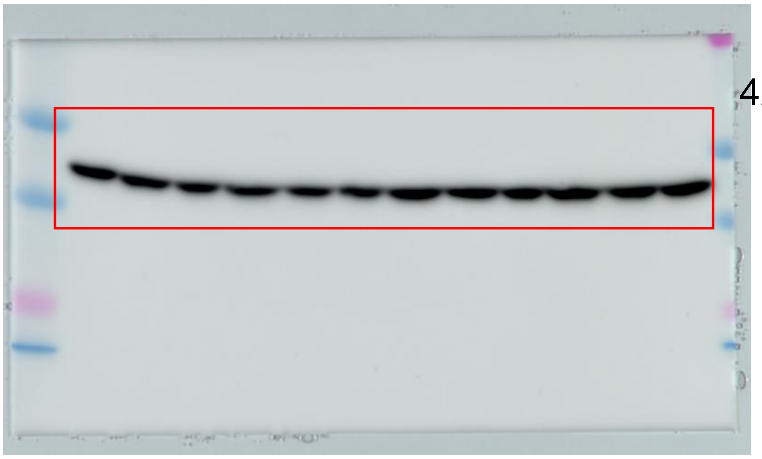
42 kDa
β actin, upper panel

E

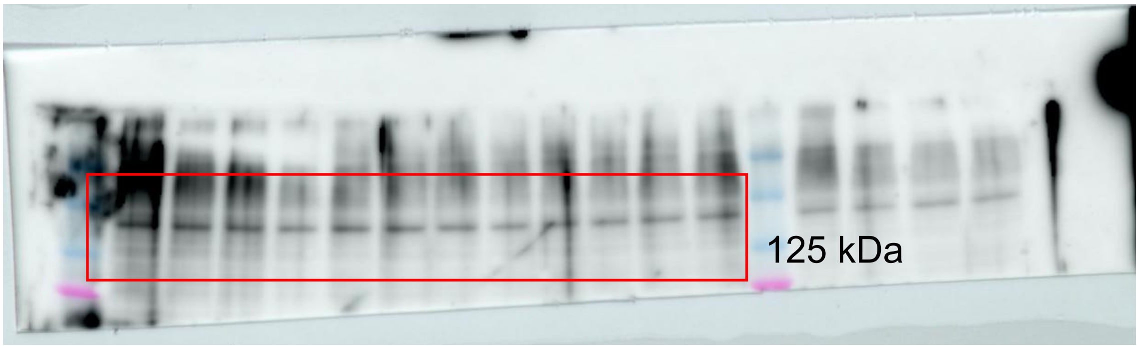
125 kDa
integrin β3

F

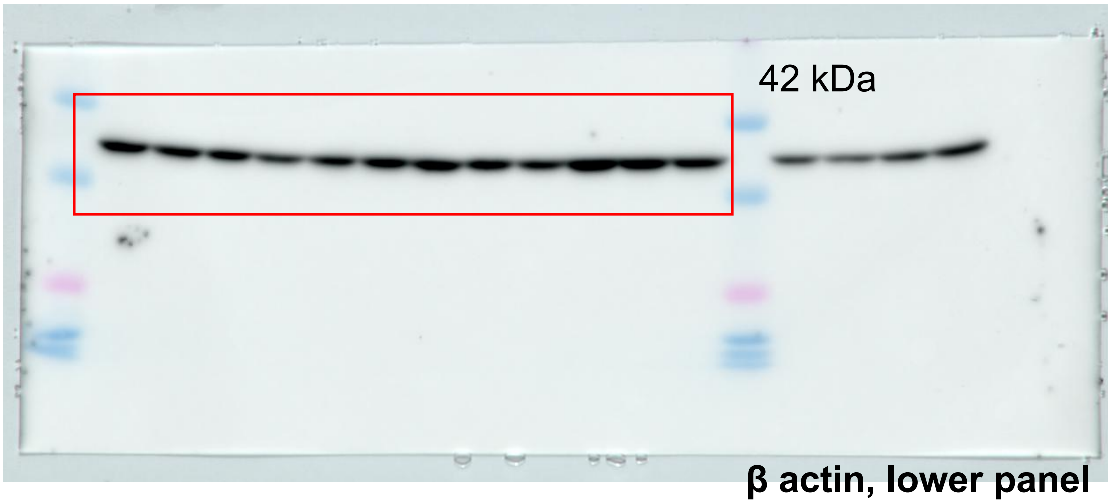
42 kDa
β actin, lower panel

G

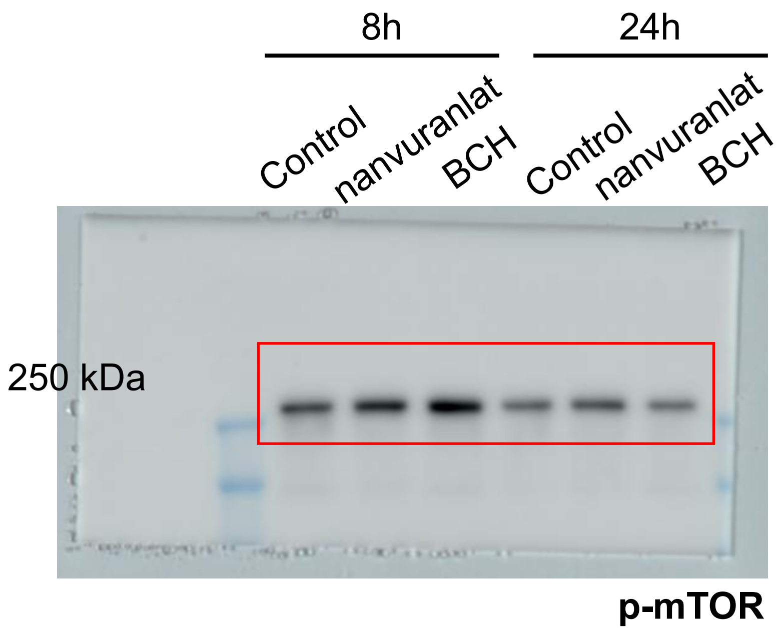
8h
24h
Control
nanvuranlat
BCH
Control
nanvuranlat
BCH
250 kDa
p-mTOR

H

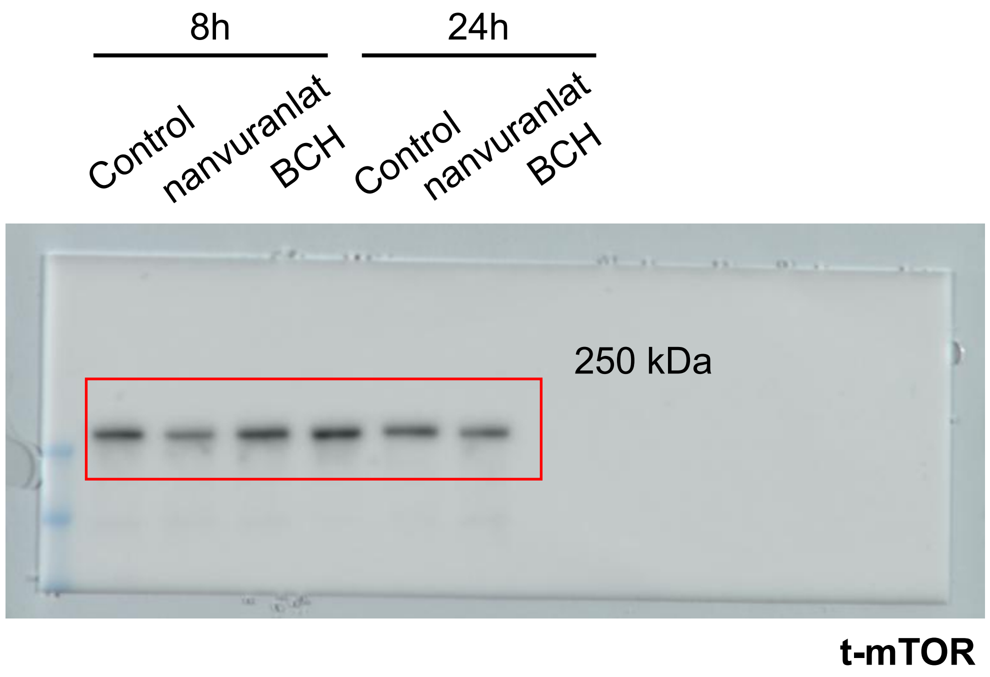
8h
24h
Control
nanvuranlat
BCH
Control
nanvuranlat
BCH
250 kDa
t-mTOR

I

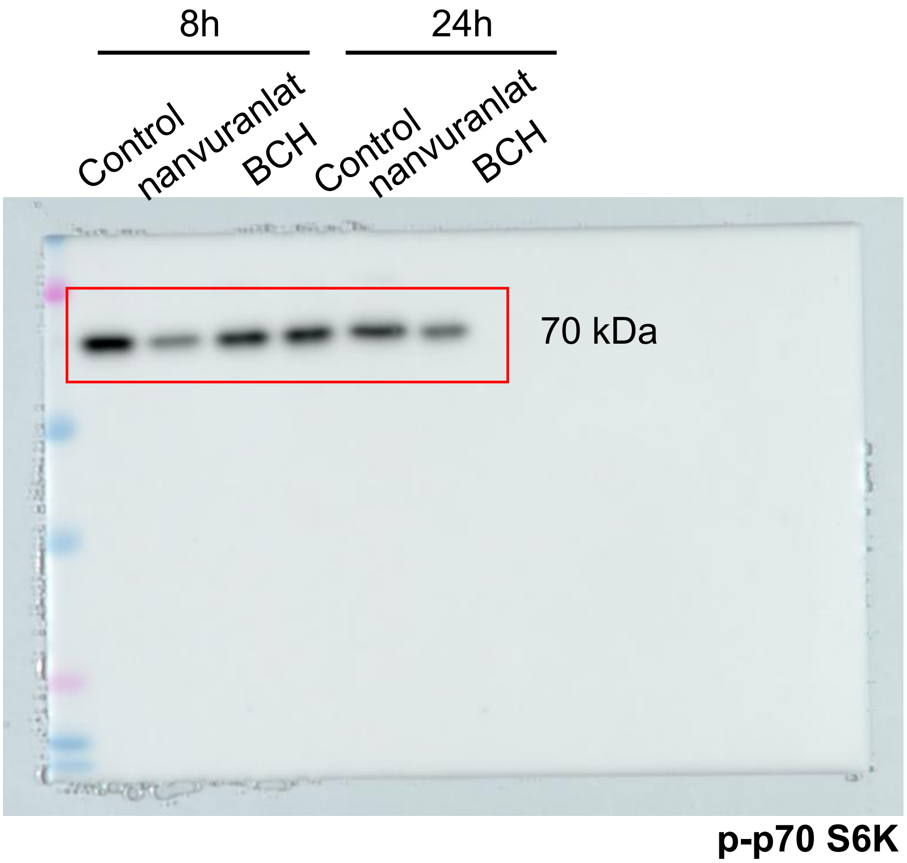
8h
24h
Control
nanvuranlat
BCH
Control
nanvuranlat
BCH
70 kDa
p-p70 S6K

J

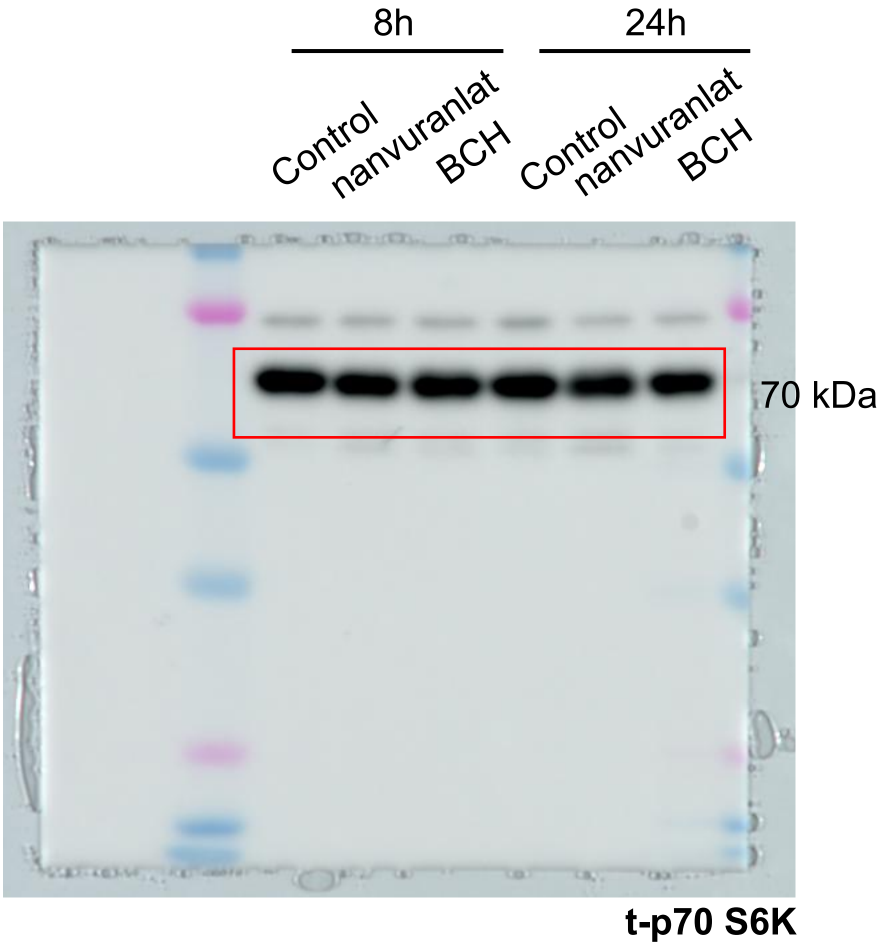
8h
24h
Control
nanvuranlat
BCH
Control
nanvuranlat
BCH
70 kDa
t-p70 S6K

K

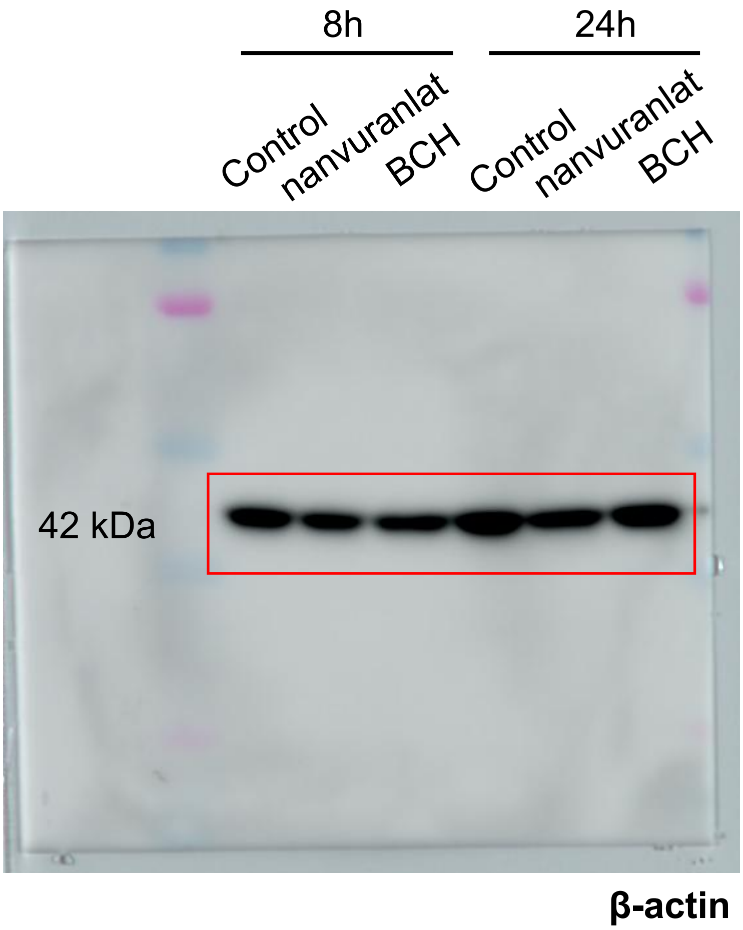

Supplementary Figure S5. Original, uncropped electrophoretic blots. ***A.*** LAT1 (Fig.1B). ***B.*** 4F2hc (Fig.1B). ***C.*** integrin αv (Fig. 7B). ***D.*** β actin (Fig. 7B, upper panel). ***E.*** integrin β3 (Fig. 7B). ***F.*** β actin (Fig. 7B, lower panel). ***G.*** p-mTOR (Fig. 7D). ***H.*** t-mTOR (Fig. 7D). ***I.*** p-p70 S6K (Fig. 7D). ***J.*** t-p70 S6K (Fig. 7D). ***K.*** β actin (Fig. 7D).
